# Pandemic paranoia in the general population: international prevalence and sociodemographic profile

**DOI:** 10.1017/S0033291722002975

**Published:** 2023-09

**Authors:** Lyn Ellett, Björn Schlier, Jessica L. Kingston, Chen Zhu, Suzanne Ho-wai So, Tania M. Lincoln, Eric M. J. Morris, Brandon A. Gaudiano

**Affiliations:** 1 School of Psychology, University of Southampton, Southampton, UK; 2 Department of Clinical Psychology and Psychotherapy, University of Hamburg, Hamburg, Germany; 3 Department of Psychology, Royal Holloway, University of London, London, UK; 4 Department of Psychology, The Chinese University of Hong Kong, Shatin, Hong Kong; 5 School of Psychology and Public Health, La Trobe University, Melbourne, Australia; 6 Department of Psychiatry and Human Behavior, Brown University, Providence, RI, USA

**Keywords:** COVID-19 pandemic, general population, international, paranoia, prevalence

## Abstract

**Background:**

The term ‘pandemic paranoia’ has been coined to refer to heightened levels of mistrust and suspicion towards other people specifically due to the COVID-19 pandemic. In this study, we examine the international prevalence of pandemic paranoia in the general population and its associated sociodemographic profile.

**Methods:**

A representative international sample of general population adults (*N* = 2510) from five sites (USA *N* = 535, Germany *N* = 516, UK *N* = 512, Australia *N* = 502 and Hong Kong *N* = 445) were recruited using stratified quota sampling (for age, sex, educational attainment) and completed the Pandemic Paranoia Scale (PPS).

**Results:**

The overall prevalence rate of pandemic paranoia was 19%, and was highest in Australia and lowest in Germany. On the subscales of the PPS, prevalence was 11% for persecutory threat, 29% for paranoid conspiracy and 37% for interpersonal mistrust. Site and general paranoia significantly predicted pandemic paranoia. Sociodemographic variables (lower age, higher population size and income, being male, employed and no migrant status) explained additional variance and significantly improved prediction of pandemic paranoia.

**Conclusions:**

Pandemic paranoia was relatively common in a representative sample of the general population across five international sites. Sociodemographic variables explained a small but significant amount of the variance in pandemic paranoia.

## Introduction

Persecutory delusions are defined as beliefs that others are intentionally trying to cause one harm, either now or in the future (Freeman & Garety, 2000). Although persecutory delusions are traditionally associated with clinical diagnoses such as schizophrenia, research also suggests that milder forms of paranoid thinking, such as ‘suspiciousness, assumptions of ill will or hostility, or notions of conspirational intent’ (Fenigstein & Vanable, 1992) are common in the general population (Bebbington et al., 2013; Ellett, Lopes, & Chadwick, 2003; Fenigstein & Vanable, 1992; Freeman et al., 2011, 2019). This is consistent with a dimensional understanding of mental health, within which an experience such as clinical paranoia is held to lie on a continuum with paranoia-like experiences seen in the general population (Strauss, 1969; Van Os, Hanssen, Bijl, & Ravelli, 2000). Paranoia has also been conceptualised as a personality trait distributed within the general population (Fenigstein & Vanable, 1992). This framing of paranoia is consistent with conceptualisations of other personality traits (see Ellett et al., 2022), such as narcissism, which have both non-clinical (general population) and clinical (narcissistic personality disorder) counterparts (Hepper, Ellett, Kerley, & Kingston, 2022).

Varying estimates of the prevalence of general paranoia have been reported in large-scale UK-based nationally representative surveys, with endorsement rates of paranoid thoughts ranging from approximately 1.5% to 18.6% in the general population (Freeman et al., 2011; Johns et al., 2004). More recently, the Revised Green Paranoid Thoughts Scale has established clinical cut-offs by specifying paranoia severity ranges, with corresponding pre-pandemic norms for clinical and non-clinical groups (Freeman et al., 2019). In the Freeman et al. study, 73% of the non-clinical population sampled scored within the average range, 27% scored in the elevated range, 15% scored in the moderately severe range, 7% in the severe range and 1% in the very severe range.

The COVID-19 pandemic has created a unique environment within which to examine paranoid thinking – it is an interpersonally threatening global context within which people may be experiencing heightened levels of mistrust towards others, and which might arguably give rise to higher levels of paranoid beliefs. Consistent with this, recent research that has examined general paranoid beliefs during the pandemic suggests that moderately severe paranoia (a score of 11+ on the R-GPTS, Freeman et al., 2019) was reported in a UK general population sample during the pandemic (Rosebrock et al., 2021), that paranoid thinking is higher than pre-established norms in Chinese students (Jiang, 2020), and that students and younger people might be especially vulnerable to heightened paranoia during the pandemic (Lopes, Bortolon, & Jaspal, 2020). Additionally, being exposed to threatening COVID-19-related stimuli (such as media coverage) has been shown to be associated with paranoia (Lopes et al., 2020), as have catastrophic beliefs about the pandemic, including thoughts of being targeted by others (Rosebrock et al., 2021). Collectively, this evidence suggests that the COVID-19 pandemic is having a negative impact on paranoid beliefs. However, research to date has focused on examining *general* paranoid beliefs during the pandemic, and studies have typically been conducted in single countries only, for example, in the UK (Rosebrock et al., 2021), Portugal (Lopes et al., 2020), USA (Larsen, Donaldson, Liew, & Mohanty, 2021) and France (Bortolon, Capdevielle, Dubrequucq, & Raffard, 2021). Furthermore, it is important to examine not only the prevalence of general paranoid beliefs, but also paranoid beliefs *specifically related to the pandemic* – what we refer to as ‘pandemic paranoia’. The term ‘pandemic paranoia’ has been coined to refer to ‘paranoid cognitions that focus specifically on the threat posed by others to oneself because of the pandemic’ (Kingston et al., 2021), for example, thinking that others are spreading rumours that you have COVID-19, or thinking that others want to infect you with COVID-19.

To examine pandemic paranoia, our research group developed and validated the Pandemic Paranoia Scale (PPS, Kingston et al., 2021). The PPS consists of 25 items and was developed to measure paranoid thoughts specifically in relation to the pandemic. It has been validated in five international sites (UK, USA, Germany, Australia and Hong Kong) and in three different languages (English, German and Chinese), using representative samples of the general population in terms of sex, age and educational attainment. A total score on the PPS can be derived (range 0–100) and there are three subscales – persecutory threat (e.g. ‘people have been hostile towards me on purpose because they think I have COVID-19’); paranoid conspiracy (e.g. ‘COVID-19 is a conspiracy to make us all feel threatened’) and interpersonal mistrust (e.g. ‘I need to be on my guard against others to protect myself from getting COVID-19’). The PPS has been shown to have very good psychometric properties, and the total score was shown to be associated with general paranoid beliefs (Kingston et al., 2021). Using the same dataset as the PPS validation study, the current paper aimed to: (1) establish the international prevalence of pandemic paranoia; (2) examine whether prevalence rates of pandemic paranoia differed across the five international sites (Australia, UK, USA, Germany and Hong Kong) and (3) determine the sociodemographic factors that predict pandemic paranoia.

## Method

### Participants

A power calculation to determine sample size for prevalence estimation from a survey was conducted. This indicated that for 95% precision and a 95% level of confidence interval, with prevalence estimated at 50% (this is recommended if prevalence is unknown as it will result in the highest possible sample size), a minimum of *N* = 385 per site was required.

Table 1 shows the sociodemographic characteristics of the sample overall and by site. In total, 2690 participants fulfilled quota and eligibility criteria; of these 2510 completed the survey and passed all five attention checks. The sample therefore consisted of 2510 participants from five international sites: United States (*N* = 535), Germany (*N* = 516), United Kingdom (*N* = 512), Australia (*N* = 502) and Hong Kong (*N* = 445). Stratified quota sampling was employed, ensuring a representative sample was recruited in each country on the basis of sex, age and educational attainment. Average age was 43.32 (±15.73), and 1323 participants (53%) were female.

**Table 1.** Sociodemographic characteristics of the sample

	Total (*n* = 2510)	UK (*n* = 512)	USA (*n* = 535)	AU (*n* = 502)	GE (*n* = 516)	HK (*n* = 445)
Age, *M* years (s.d.)	43.32 (15.73)	41.91 (14.87)	47.65 (17.05)	44.75 (17.55)	42.00 (13.79)	39.64 (13.57)
Gender (%)						
Male	46.9%	47.1%	46.4%	48.2%	49.2%	43.1%
Female	52.5%	52.7%	52.7%	50.8%	50.0%	56.6%
Genderqueer	0.2%	0%	0.2%	0.2%	0.6%	0%
Transmale/female	0.1%	0%	0.4%	0.2%	0.2%	0.2%
Other	0.2%	0%	0.4%	0.4%	0%	0%
Education						
Primary	1.9%	0.4%	5.2%	0.8%	0.4%	2.5%
Secondary or equivalent	24.5%	19.7%	0%	15.5%	59.7%	28.8%
A-level or equivalent	30.8%	38.3%	34.4%	49.2%	12.8%	18.2%
Bachelor degree or equivalent	31.3%	30.3%	46.7%	28.9%	11.4%	39.8%
Master's degree or equivalent	10.0%	9.4%	11.0%	4.6%	14.5%	10.1%
Ph.D. or equivalent	1.5%	2.0%	2.6%	1.0%	1.2%	0.7%
Income						
Under £18 500	19.3%	15.6%	26.7%	22.9%	20.9%	8.5%
£18 500–£36 999	28.6%	39.8%	25%	27.1%	28.3%	22.2%
£37 000–£55 999	20.8%	23.6%	16.1%	13.3%	23.4%	28.8%
£56 000–£74 999	12.3%	11.5%	10.1%	13.3%	14.7%	11.7%
£75 000–£92 999	8.6%	4.7%	6.9%	12.4%	6.2%	13.9%
£93 000–£111 999	5.7%	2.1%	7.5%	7.4%	3.3%	8.3%
£112 000+	4.7%	2.5%	7.7%	3.6%	3.1%	6.5%
Employment						
Full time	50.9%	50.4%	40.9%	41.8%	50.2%	74.4%
Part time	14.2%	20.7%	8.8%	13.9%	17.6%	9.7%
Retired	7.9%	10.4%	0%	16.9%	8.7%	3.6%
Unemployed	11.2%	6.9%	27.0%	10.2%	7.9%	2.3%
Military	0.4%	0%	0%	0%	0.2%	0%
Home keeper/carer	5.7%	5.7%	9.2%	7.2%	4.5%	1.3%
Disabled	3.0%	1.6%	4.7%	6.0%	2.5%	0%
Training/school	6.7%	4.3%	8.2%	4.0%	8.3%	8.8%

### Measures

*Pandemic Paranoia Scale* (*PPS*, Kingston et al., 2021) is a 25-item measure that assesses paranoid thinking focused specifically on the threat posed from others to oneself because of the COVID-19 pandemic. Each item is rated on a 0 (‘not at all’) to 4 (‘totally’) scale, with total scores ranging from 0 to 100. There are three subscales that assess persecutory threat (15 items), paranoid conspiracy (six items) and interpersonal mistrust (four items). Internal consistency was acceptable for the total score on the scale (*α* = 0.71), and good to excellent on the three subscales (persecutory threat *α* = 0.97, paranoid conspiracy *α* = 0.93 and interpersonal mistrust *α* = 0.83). Good test-retest reliability and construct validity have also been established (Kingston et al., 2021). PPS items were translated into German and Chinese (see Kingston et al., 2021 for details). The full list of all PPS items can be found in online Supplementary material 1.

### Procedure

Ethical approval was obtained in each of the five international sites. Potential participants were contacted by Qualtrics (an online survey software platform) to participate in the study, and all provided informed consent. Participants completed the questionnaires online between February and March 2021. To ensure data accuracy, five attention checks were embedded throughout the survey, and participants who took less than half of the median time to complete the survey were excluded. All participants were reimbursed for taking part.

### Statistical analyses

Analyses were conducted using SPSS version 24 for Windows, Jamovi version 1.8.1.0 for Windows (see https://www.jamovi.org), and R version 4.1.0. Frequency distributions using R-GPTS cut-off scores were used to examine general paranoia.

To establish the prevalence of pandemic paranoia, frequency distributions of item endorsement of total PPS scores and the three subscales were calculated. Endorsement was operationalised as items with a rating of scale-midpoint (i.e. 2) or above (range 0–4, thus endorsement mirrored some level of agreement/absence of disagreeing with the item-content). Endorsement rate was calculated as the percentage of endorsed items on the total PPS and on each subscale. Kruskal–Wallis tests were used to compare endorsement rate and PPS total scores across sites, given the non-normality of the raw scores. It needs noting that while our choice of midpoint-dichotomisation is consistent with prior research (e.g. Bird, Evans, Waite, Loe, and Freeman, 2016; Freeman et al., 2005), both more liberal (i.e. endorsement being operationalised as any value above the lowest item score; Delespaul, devries, & van Os, 2002; Schlier, Winkler, Jaya, & Lincoln, 2018) and more restrictive dichotomisation criteria (e.g. endorsement above the midpoint; Kuhn, Lieb, Freeman, Andreou, & Zander-Schellenberg, 2021) have also been used when measuring psychosis symptoms/conspiracy beliefs in different samples. To provide a balanced view and avoid presenting an inflated estimate of pandemic paranoia, we chose the midpoint dichotomisation as our main analysis, but also added a detailed overview of extreme endorsement only (i.e. 4) in online Supplementary material to this article. Additionally, we tested whether differences in endorsement rates reflect true differences in pandemic paranoia since the prior validation showed configural and metric invariance, but not scalar invariance for the PPS (Kingston et al., 2021). To do so, we estimated latent trait values for the PPS subscales in a confirmatory factor analysis environment using the projection method in the R-package *equaltestMI* (Jiang, Mai, & Yuan, 2017). In this approach, the observed values of the manifest variables (i.e. items) are decomposed into the two orthogonal components (1) common scores and (2) specific factors. This permits testing for equality of the common latent trait means across groups (i.e. sites) independently from (site-)specific factors even when only the constrain of equal loadings (metric invariance), but not the constrain of equal intercepts (scalar invariance) is met (Jiang et al., 2017). Specifically, we calculated and tested for common factor differences (that reflect true latent mean differences between sites) by calculating latent trait models for pairwise comparisons using subsamples from two sites, respectively. For each pairwise comparison, we calculated two models: a unifactorial model for testing the PPS global score differences and a three factorial model for the three subscales, using the raw item scores (0–4) as manifest variables.

Lastly, to examine the sociodemographic profile of pandemic paranoia, we calculated Pearson correlations (for continuous sociodemographic predictors) and Cohen's *d* (for dichotomous predictors) within each site to establish site-specific demographic profiles. Next, we integrated the results using random-effects models to average a general profile. Additionally, we used stepwise linear regression to test the effects of global and site-specific sociodemographic factors after controlling for the effects of site and general paranoia.

## Results

### International prevalence of pandemic paranoia

Descriptive statistics on the total PPS and for each PPS subscale for the total sample and for each site are shown in Table 2. The mean total score on the PPS in the full sample was 15.86 (s.d. = 18.20, range 0–100). Mean scores on the subscales were: persecutory threat (*M* = 5.34, s.d. = 11.24); paranoid conspiracy (*M* = 5.69, s.d. = 6.61) and interpersonal mistrust (*M* = 4.82, s.d. = 4.28). Details of endorsement rates by item and more conservative endorsement estimates based on endpoint answers on the Likert-scales only (total score mean endorsement rates: 1.74–8.29%) can be found in online Supplementary material 1.

**Table 2.** Descriptive statistics on pandemic paranoia for the total sample and by site

	PPS total		Persecutory threat		Paranoid conspiracy		Interpersonal mistrust	
	*M* (s.d.)	Endorse %	*M* (s.d.)	Endorse %	*M* (s.d.)	Endorse %	*M* (s.d.)	Endorse %
Total	15.86 (18.20)	19.44	5.34 (11.24)	11.11	5.69 (6.61)	28.60	4.82 (4.28)	36.93
UK	12.52 (14.41)	14.80	2.94 (8.24)	5.69	4.40 (5.77)	21.26	5.18 (4.34)	39.26
USA	15.88 (19.93)	19.65	5.55 (12.56)	11.83	5.63 (7.17)	27.98	4.70 (4.64)	36.50
Australia	20.58 (22.20)	25.71	8.47 (12.30)	18.05	6.59 (7.10)	33.76	5.52 (4.37)	42.38
Germany	12.49 (14.42)	15.23	3.71 (8.36)	7.64	4.85 (6.21)	24.64	3.93 (4.04)	29.60
Hong Kong	18.24 (17.35)	22.32	6.23 (10.61)	12.67	7.22 (6.41)	36.55	4.79 (3.70)	37.13

*Note*: Endorse %, endorsement rate; PPS, Pandemic Paranoia Scale.

**Fig. 1.**
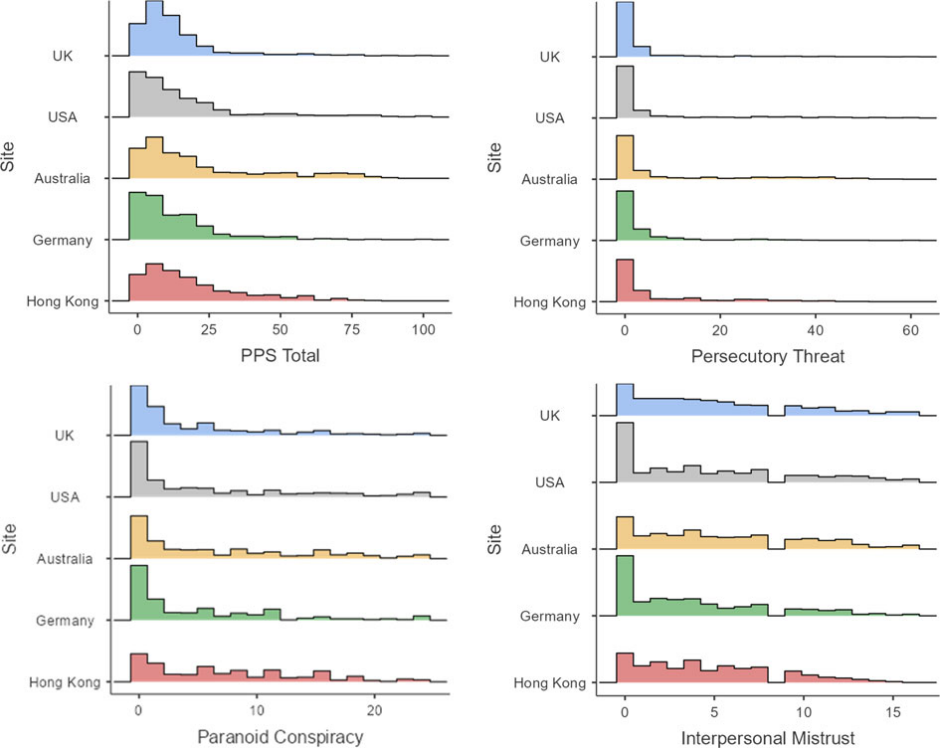
Sum scores distribution by site for Pandemic Paranoia Scale total and subscales.

The distribution of scores on the PPS and three subscales across all five sites are shown in Fig. 1. Pandemic paranoid thoughts were commonly endorsed by participants, with monthly occurrence by item ranging from 9% (‘people are spreading the rumour that I have COVID-19’) to 48% (‘I can't trust others to stick to the social distancing rules’). The mean number of pandemic paranoid thoughts endorsed across the total sample was 4.86 (s.d. = 6.32, range 0–25).

Across the whole sample, the average endorsement rate was 19% for the PPS total score. The most commonly endorsed subscale of the PPS was interpersonal mistrust (average endorsement 36%), followed by paranoid conspiracy (average endorsement 29%), and persecutory threat was the least endorsed subscale with an average endorsement rate of 11%. Correspondingly, we found mean scores for the persecutory threat scale to be significantly lower than both paranoid conspiracy (−0.79 ⩽ *M_differences_* ⩽ −0.53) and interpersonal mistrust (−1.10 ⩽ *M_differences_* ⩽ −0.74) in each of the five sites in pairwise *t* tests (all *p*'s <0.001).

### Differences in prevalence of pandemic paranoia by international site

There was an effect of site on the average number of PPS items endorsed [χ^2^(4) = 41.89, *p* < 0.001]. Post-hoc pairwise comparisons showed that the UK, USA and Germany all endorsed significantly fewer items than both Australia and Hong Kong. There was no difference in number of items endorsed between Australia and Hong Kong. Kruskal–Wallis tests revealed significant differences between the sites on the PPS total score [χ^2^(4) = 41.89, *p* < 0.001], and all three subscales [persecutory threat: χ^2^(4) = 50.54, *p* < 0.001; paranoid conspiracy χ^2^(4) = 61.05, *p* < 0.001; interpersonal mistrust χ^2^(4) = 37.16, *p* < 0.001]. Pairwise comparisons by site are shown in Table 3. Australia scored significantly higher than the UK, USA and Germany on PPS total scores. Additionally, Hong Kong scored significantly higher than both the UK and Germany on the PPS total score. Differential patterns emerged for the three subscales. On the persecutory threat subscale, the UK scored significantly lower compared to all other sites, and Australia was significantly higher than both the USA and Germany. On the paranoid conspiracy subscale, the UK, USA and Germany were all significantly lower than both Australia and Hong Kong. On the interpersonal mistrust subscale, the UK, USA, Australia and Hong Kong were all higher than Germany.

**Table 3.** Site pairwise comparisons on the PPS total and subscales

		PPS total		Persecutory threat		Paranoid conspiracy		Interpersonal mistrust	
		*W*	diff_Latent_	*W*	diff_Latent_	*W*	diff_Latent_	*W*	diff_Latent_
UK	USA	1.81	**0**.15***	**4**.47**	**0**.16***	3.05	**0**.22**	−2.21	0.07
UK	Australia	6.67***	0.33***	9.19***	0.34***	7.52***	0.40***	1.77	0.08
UK	Germany	−0.50	0.01	5.14**	0.04	1.88	0.09	−6.53***	−0.35***
UK	Hong Kong	5.84***	0.21***	7.86***	0.19***	9.45***	0.52***	−1.15	−0.10
USA	Australia	4.64**	0.20***	4.76**	0.19***	4.26*	0.17*	3.86	0.20**
USA	Germany	−2.23	−0.14**	0.42	−0.12**	−1.37	−0.14	−4.11*	−0.21**
USA	Hong Kong	3.76	0.09	3.33	0.04	6.01***	0.29***	1.04	0.02
Australia	Germany	−7.03***	−0.32***	−4.81**	−0.29***	−5.70***	−0.32***	−8.06***	−0.42***
Australia	Hong Kong	−0.98	−0.10*	−1.64	−0.14**	1.88	0.12	−2.87	−0.17**
Germany	Hong Kong	6.12***	0.20***	3.22	0.14***	7.58***	0.45***	5.31**	0.22**

*Note*: Dwass-Steel-Critchlow-Fligner pairwise *W* statistics are reported. PPS, Pandemic Paranoia Scale. diff_Latent_, Differences between estimated latent trait means in a projection based analysis

Significant differences are printed in bold. *p < 0.050, **p < 0.010, ***p < 0.001.

Most of these results could also be found in the analysis of latent mean differences. However, the difference in persecutory threat scores between UK and Germany did not extend to the latent trait. Furthermore, latent mean analysis also showed additional differences that were occluded in PPS score comparison: The USA scored significantly higher latent traits in the total score than the UK and Germany, which extended to a higher latent trait paranoid conspiracy than the UK and a higher latent trait in persecutory threat than Germany. Regarding interpersonal mistrust, the USA showed a significantly lower latent mean than Australia. Similarly, additional significant differences for the Hong Kong sample showed significantly lower latent means in the total score, persecutory threat and interpersonal mistrust than Australia and a significantly higher persecutory threat latent mean than Germany. Overall, equivalence testing showed that for the subscales, manifest differences in items reflected 46.1% (USA *v.* Germany) to 93.7% (UK *v.* Australia) of true latent mean differences in the pairwise comparisons, whereas the range of true latent mean difference reflected in the total score ranged from 0.4% (UK *v.* Germany) to 92.3% (UK *v.* Australia).

### Sociodemographic profile of pandemic paranoia

Integration of effect sizes (correlation/Cohen's *d*) for total PPS score and sociodemographic variables yielded a global significant effect for age [*r* = −0.24, *z* = −6.59, *p* < 0.001, 95% CI (−0.30 to −0.17)]. Integrated effects for income (*r* = 0.18, *z* = 1.87, *p* = 0.061), education (*r* = 0.11, *z* = 1.67, *p* = 0.096) and population size (*r* = 0.15, *z* = 1.94, *p* = 0.052) were not significant. Table 4 provides an overview of the effects by site. As can be seen, age correlated significantly with PPS scores in all five sites. Regarding the other demographic variables, there was considerable variability, with Australia and the USA showing a sociodemographic profile with more significant predictors and stronger effects, the UK showing multiple significant sociodemographic predictors albeit with weaker effects, and Germany and Hong Kong showing no significant predictor beyond age. Higher pandemic paranoia scores correlated with higher income, higher education, being employed, being male and no migrant status.

**Table 4.** Global and site-specific association between pandemic paranoia (PPS sum score) sociodemographic variables

	Integrated effect					UK		USA		Australia		Germany		Hong Kong	
	*r*/*d*	*Z*	95% CI		*I*^2^ (%)	*r* or *d*	*p*	*r* or *d*	*p*	*r* or *d*	*p*	*r* or *d*	*p*	*r* or *d*	*p*
Age	***r* = −0.237**	**−6.59**	**−0.304**	**−0.169**	70	***r* = −0.220**	<0.001	***r* = −0.320**	<0.001	***r* = −0.272**	<0.001	***r* = −0.258**	<0.001	***r* = −0.106**	0.029
Income	*r* = 0.181	1.94	−0.008	0.358	96	***r* = 0.122**	0.006	***r* = 0.303**	<0.001	***r* = 0.459**	<0.001	*r* = −0.017	0.705	*r* = −0.001	0.981
Education	*r* = 0.111	1.67	−0.020	0.237	91	***r* = 0.106**	0.017	***r* = 0.214**	<0.001	***r* = 0.291**	<0.001	*r* = −0.047	0.286	*r* = −0.022	0.647
Population size	*r* = 0.149	1.67	−0.001	0.294	93	***r* = 0.177**	<0.001	***r* = 0.382**	<0.001	***r* = 0.192**	<0.001	*r* = 0.010	0.828	*r* = −0.031	0.521
Male sex	*d* = 0.261	1.55	−0.068	0.590	94	*d* = −0.001	0.991	***d* = 0.510**	<0.001	***d* = 0.804**	<0.001	*d* = 0.085	0.332	*d* = −0.094	0.328
Unemployed	*d* = −0.180	−1.04	−0.520	0.160	83	*d* = −0.094	0.591	***d* = −0.508**	<0.001	***d* = −0.594**	<0.001	*d* = 0.195	0.232	*d* = 0.340	0.289
Migration	*d* = −0.135	−1.18	−0.333	0.083	54	*d* = 0.123	0.353	*d* = −0.193	0.313	***d* = −0.390**	0.001	*d* = −0.009	0.956	*d* = −0.144	0.494

*Note*: Pearson correlation or Cohen's *d* and independent groups *t* tests were calculated for the associations in each individual site. Results from the five sites were integrated with a random-effects meta-analysis. Significant results are printed in bold.

A stepwise multiple regression analysis was conducted, with the PPS total score as the dependent variable. To control for site-level differences and general paranoia score, site and R-GPTS scores were added in block one. Sociodemographic variables added in block two, and interaction effects of sociodemographic variables and site added in step three. Site and general paranoia accounted for 47.42% of the variance in pandemic paranoia and collectively predicted it significantly [*F*_(2, 2270)_ = 409.5, *p* < 0.001]. The sociodemographic variables explained an additional 2.39% (*R*^2^ = 0.498) of the variance and significantly improved prediction of pandemic paranoia [*F*_(7, 2263)_ = 15.99, *p* < 0.001]. Lower age (*β* = −0.03, *t* = −1.98, *p* = 0.048), higher average income (*β* = 0.08, *t* = 4.66, *p* < 0.001), higher population size (*β* = 0.06, *t* = 3.23, *p* = 0.001), being male (*β* = −0.06, *t* = −4.22, *p* < 0.001), employed (*β* = −0.03, *t* = −2.13, *p* = 0.033) and no migrant status (*β* = −0.03, *t* = −2.10, *p* = 0.033) all significantly predicted more pandemic paranoia. Adding the site by demographic variable interaction effects (with site dummy coded and UK set to 0) in the final step significantly increased the explained variance by another 2.61% [*R*^2^ = 0.524, *F*_(28, 2235)_ = 4.38, *p* < 0.001]. Significant interactions were found for the Australian site by (1) income (*β* = 0.20, *t* = 3.92, *p* = 0.001), (2) sex (*β* = −0.07, *t* = −2.30, *p* = 0.022) and (3) migrant status (*β* = −0.06, *t* = −2.67, *p* = 0.008).

## Discussion

This study examined the international prevalence and sociodemographic profile of pandemic paranoia in a large representative sample of the general population in five international sites. Across the total sample, pandemic paranoia was relatively common, with an overall prevalence rate of 19%. For the PPS subscales, prevalence was 11% for persecutory threat, 29% for paranoid conspiracy and 37% for interpersonal mistrust. These findings suggest that interpersonal mistrust was the most common, and persecutory threat was the least common facet of pandemic paranoia. Prevalence rates of pandemic paranoia, particularly the interpersonal mistrust facet, are also broadly consistent with recent meta-analytic findings of the prevalence of other mental health indices during the COVID-19 pandemic, including anxiety (31.9%) and depression (33.7%) (Salari et al., 2020). Longitudinal data are now needed to track the prevalence of pandemic paranoia over time and determine whether it is stable and/or clinically significant.

Some interesting differences in the prevalence of pandemic paranoia between the five international sites also emerged. Notably, Australia had the highest overall prevalence of pandemic paranoia, and Germany reported the lowest prevalence. In relation to the individual PPS subscales: the UK had the lowest levels of persecutory threat; the UK, USA and Germany had significantly lower levels of paranoid conspiracy compared with Australia and Hong Kong; and Germany had the lowest level of interpersonal mistrust. Although it is not possible to draw any definitive conclusions regarding causal effects of these site-level differences in prevalence, we can explore some possible explanations. First, we considered whether the incidence of COVID-19 cases in the five sites at the time of data collection may have impacted on our findings. However, as the cumulative confirmed case count was second lowest in Australia (28 823) (John Hopkins University, 2020; Roser, Ritchie, Ortiz-Ospina, & Hasell, 2020), and pandemic paranoia was highest in this site, this explanation seems unlikely. Second, other pandemic-specific contextual factors may also potentially be relevant, such as different social distancing and lockdown rules across sites, as well as potential differences in availability of vaccines during data collection. For example, the extensive lockdown in Australia could explain the high rate of pandemic paranoia found. Additionally, in the USA, lockdown rules and mask-wearing practices differed significantly between states/regions, such that there could also have been within-site differences in paranoid experiences and pandemic paranoia. Furthermore, cross-cultural factors could also account for site-level differences in prevalence, though they were not measured in the current study. It will therefore be important in future research to establish the causal mechanisms of pandemic paranoia.

In relation to the sociodemographic profile of pandemic paranoia, the only variable to show a consistent relationship with pandemic paranoia across all five sites was age. Findings suggest that lower age was associated with higher PPS scores, suggesting that younger people might be especially vulnerable to experiencing pandemic paranoia. This finding aligns with previous research showing an association between younger age and general paranoia both pre-pandemic (Freeman et al., 2011) and during the pandemic (Bortolon et al., 2021; Lopes et al., 2020). Age is clearly emerging as a robust predictor of paranoia. Additionally, we found that after controlling for site and general paranoia, sociodemographic variables collectively explained an additional small (2.39%) but significant amount of variance in total PPS scores. Lower age, higher population size and income, being male, employed and having no migrant status all significantly predicted pandemic paranoia. These findings are consistent with previous research, for example, showing that the highest levels of paranoia are more common in men (Freeman et al., 2011) and that general paranoia is associated with urban dwelling (e.g. Ellett, Freeman, & Garety, 2008; van Os, 2004). There are also, however, some inconsistencies, in that general paranoia is typically associated with lower socio-economic status and migrant status (e.g. Freeman et al., 2011), and here we found that pandemic paranoia was associated with higher income and no migrant status. The impact of the pandemic on work and school settings might help to explain this. There are limited data comparing sociodemographic profiles of general paranoia across different cultures and countries, which makes interpretation of our findings in relation to pandemic paranoia more challenging. Clearly, there is heterogeneity in findings relating to sociodemographic predictors of paranoia that warrant further consideration.

The study has some limitations. First, we used a cross-sectional survey design, and although stratified quota sampling was employed to recruit a representative general population sample in all five international sites, no conclusions about causality can be made, and the findings are limited in terms of generalisability to individuals with clinical paranoia. It would be interesting in future research to examine the prevalence of pandemic paranoia in clinical samples and assess its stability in longitudinal research. Second, we relied solely on self-report measures, which could have introduced bias. Third, we collected data in high income countries only, and future research might usefully examine pandemic paranoia in middle- and low-income countries. Fourth, the study raises an interesting conceptual issue about what is considered to be ‘paranoia’ as a threshold between its clinical and non-clinical counterparts. Unfortunately, we were not able to establish clinical cut offs for the PPS as it is a new measure, but note that recent research has established clinical cut offs for general paranoia (Freeman et al., 2019). Fifth, although it could be argued that choosing 2 as a cut-off for calculating PPS prevalence (item scale 0–4) is arbitrary, we chose the midpoint dichotomisation as our main analysis to provide a balanced view and to avoid presenting an inflated estimate of pandemic paranoia. We refer interested readers to online Supplementary material 1, where we present a detailed overview of extreme endorsement only (i.e. 4). Finally, we did not measure any belief dimensions associated with pandemic paranoia, such as conviction or distress, which would be interesting to investigate in future research, given their importance in psychological interventions for paranoia (Chadwick et al., 1996).

In conclusion, our study shows that pandemic paranoia is relatively common in an international sample with an overall 19% prevalence rate, and our data suggest that pandemic paranoia is hierarchically organised. Pandemic paranoia was highest in Australia and lowest in Germany, though reasons for these site-level differences are yet to be established. Our findings also indicate that sociodemographic variables – lower age, higher population size and income, being male, employed and having no migrant status – all predicted higher levels of pandemic paranoia. The findings contribute to the growing understanding of the psychological impact of the pandemic across the globe.

## Data Availability

The UK dataset is available – https://osf.io/8jtgv/.

## Supplementary material

For supplementary material accompanying this paper visit https://doi.org/10.1017/S0033291722002975. click here to view supplementary material
