# Supplementary material for: Pandemic paranoia in the general population: international prevalence and sociodemographic profile
Source: Psychol Med. 2022 Sep 6;53(12):5748–55. doi: 10.1017/S0033291722002975 (PMC9530376; doi:10.1017/S0033291722002975)
Supplement: Supplementary file 1 [file S0033291722002975sup001.docx]

**Supplement S1.**

**Comparison of midpoint-based endorsement rates (i.e. items are considered endorsed when participants rated them with 2 or more on the 0-4 Likert scale) and endpoint-based endorsement rates (i.e. items are considered endorsed when participants rated them with 4 on the 0-4 Likert scale)**

| Table S1 Pandemic Paranoia Scale (PPS) item list and corresponding endorsement rates (total sample) | | |
| --- | --- | --- |
| PPS Item | Midpoint Endorse % | Endpoint Endorse % |
| **Subscale: Persecutory Threat** |  |  |
| I was sure someone wanted to infect me with COVID-19. | 10.08% | 1.43% |
| People are deliberately trying to pass COVID-19 to me. | 9.20% | 1.35% |
| I was certain that people did things to put me at risk of catching COVID-19. | 14.90% | 2.07% |
| People are spreading the rumour that I have COVID-19. | 9.08% | 1.39% |
| I was convinced there was a conspiracy to get me to catch COVID-19. | 11.47% | 1.71% |
| I couldn’t stop thinking about people wanting to infect me with COVID-19. | 9.88% | 1.35% |
| I was distressed by being targeted by people who wanted me to catch COVID-19. | 9.68% | 1.71% |
| I can’t stop worrying about other people spreading the rumour that I have COVID-19 | 10.28% | 1.67% |
| People have been hostile towards me on purpose because they think I have COVID-19. | 10.12% | 1.47% |
| People are watching me more closely due to COVID-19. | 14.22% | 2.51% |
| Other people are trying to harm me on purpose by not abiding to social distancing rules. | 13.03% | 2.07% |
| Strangers and friends look at me critically because they think I have COVID-19. | 10.56% | 1.63% |
| Some people try to make it hard for me to get access to face coverings and other COVID-19 protective gear. | 11.31% | 1.95% |
| People have tried to contaminate my face mask or other COVID-19 protective gear. | 9.96% | 1.71% |
| I feel threatened by people watching me more closely due to COVID-19. | 12.83% | 2.07% |
| **Subscale: Paranoid Conspiracy** |  |  |
| The government is lying to us about COVID-19. | 32.23% | 7.65% |
| The government is using the COVID-19 pandemic to control us. | 29.64% | 8.41% |
| The government is deciding things about COVID-19 behind our backs. | 38.76% | 9.88% |
| COVID-19 is a conspiracy to make us all feel threatened. | 22.59% | 5.50% |
| COVID-19 is a conspiracy by powerful people. | 20.68% | 5.42% |
| Social distancing is a way to keep people under control by the government. | 27.69% | 7.37% |
| **Subscale: Interpersonal Mistrust** |  |  |
| I need to be on my guard against others to protect myself from getting COVID-19. | 35.26% | 9.00% |
| I can’t trust others to stick to the social distancing rules. | 48.25% | 11.79% |
| I can’t stop worrying about other people failing to stick to the rules. | 31.55% | 5.98% |
| Other people cannot be trusted to keep our community safe from COVID-19. | 32.67% | 6.37% |

| Table S2. Comparison of endorsement rates (midpoint-based and extreme rating based) on pandemic paranoia for the total sample and by site. | | | | | | | | |  |
| --- | --- | --- | --- | --- | --- | --- | --- | --- | --- |
|  | PPS Total | | Persecutory Threat | | Paranoid Conspiracy | | Interpersonal Mistrust | | |
|  | midpoint  Endorse % | endpoint  Endorse % | midpoint  Endorse % | endpoint  Endorse % | midpoint  Endorse % | endpoint  Endorse % | midpoint  Endorse % | endpoint  Endorse % | |
| Total | 19.44 | 4.14 | 11.11 | 1.74 | 28.60 | 7.37 | 36.93 | 8.29 | |
| UK | 14.80 | 3.25 | 5.69 | 1.00 | 21.26 | 4.79 | 39.26 | 9.38 | |
| USA | 19.65 | 5.56 | 11.83 | 2.57 | 27.98 | 9.56 | 36.50 | 10.79 | |
| Australia | 25.71 | 5.46 | 18.05 | 3.00 | 33.76 | 8.73 | 42.38 | 9.76 | |
| Germany | 15.23 | 3.22 | 7.64 | 1.14 | 24.64 | 6.49 | 29.60 | 6.15 | |
| Hong Kong | 22.32 | 3.03 | 12.67 | 0.88 | 36.55 | 7.19 | 37.13 | 4.83 | |
| *Note.* Midpoint Endorse %, endorsement rate based on ratings of 2 or more on the 0-4 Likert scale. Endpoint Endorse %, endorsement rate based on ratings of 4 on the 0-4 Likert scale. PPS, Pandemic Paranoia Scale. | | | | | | | | |  |
